# Biomechanics in liver regeneration after partial hepatectomy

**DOI:** 10.3389/fbioe.2023.1165651

**Published:** 2023-05-05

**Authors:** Yi Wu, Ning Li, Xinyu Shu, Wang Li, Xiaoyu Zhang, Dongyuan Lü, Mian Long

**Affiliations:** ^1^ Center for Biomechanics and Bioengineering, Beijing Key Laboratory of Engineered Construction and Mechanobiology and Key Laboratory of Microgravity (National Microgravity Laboratory), Institute of Mechanics, Chinese Academy of Sciences, Beijing, China; ^2^ School of Engineering Sciences, University of Chinese Academy of Sciences, Beijing, China

**Keywords:** liver regeneration, partial hepatectomy, hemodynamics, mechanical loading, mechanotransduction

## Abstract

The liver is a complicated organ within the body that performs wide-ranging and vital functions and also has a unique regenerative capacity after hepatic tissue injury and cell loss. Liver regeneration from acute injury is always beneficial and has been extensively studied. Experimental models including partial hepatectomy (PHx) reveal that extracellular and intracellular signaling pathways can help the liver recover to its equivalent size and weight prior to an injury. In this process, mechanical cues possess immediate and drastic changes in liver regeneration after PHx and also serve as main triggering factors and significant driving forces. This review summarized the biomechanics progress in liver regeneration after PHx, mainly focusing on PHx-based hemodynamics changes in liver regeneration and the decoupling of mechanical forces in hepatic sinusoids including shear stress, mechanical stretch, blood pressure, and tissue stiffness. Also discussed were the potential mechanosensors, mechanotransductive pathways, and mechanocrine responses under varied mechanical loading *in vitro*. Further elucidating these mechanical concepts in liver regeneration helps establish a comprehensive understanding of the biochemical factors and mechanical cues in this process. Proper adjustment of mechanical loading within the liver might preserve and restore liver functions in clinical settings, serving as an effective therapy for liver injury and diseases.

## 1 Introduction

The liver is the largest substantive organ in the mammalian body and undertakes many key physiological activities within the body. It is mainly responsible for the absorption, storage, metabolism, and redistribution of substances including sugars, lipids, sterols, proteins, and vitamins. The liver also has functions in immune regulation and defensive response, detoxification, biotransformation, and bile synthesis and secretion (Michalopoulos, 2007; Powell et al., 2021). As a unique organ, the liver has a powerful regenerative capability to guarantee its mass, structure, and stability of broad hepatic functions (Michalopoulos, 2007). Recently, liver-related diseases such as hepatitis, fatty liver, cirrhosis, and liver cancer have become one of the main causes of death worldwide (Sallberg and Pasetto, 2020; Powell et al., 2021). The resection of liver lesions is a vital treatment and effectively exploring the potential of liver regeneration is the key to the survival of postoperative patients. Living donor liver transplantation utilizes the ability of liver regeneration to alleviate the problem of recipient insufficiency and donor shortage. However, challenges still remain in keeping the liver outside the body and maintaining its metabolic activity (Giwa et al., 2017; de Vries et al., 2019). Therefore, understanding the regeneration mechanisms of the liver yields both biological significance and potential applications for the treatment of liver injury and diseases (Campana et al., 2021).

Liver regeneration refers to the process of proliferation, migration, and differentiation of various hepatic cells by the combined regulation of various factors to restore the normal size and function of the liver. In contrast to the regrowth of limbs in lower vertebrates containing stem cells at the cut surface, the resected parts of the liver do not grow back from the cut surface, but instead, expand the tissue mass from the remaining lobes to compensate for the lost tissues (Fausto et al., 2006; Cordero-Espinoza and Huch, 2018), suggesting that liver regeneration is a compensatory process of liver growth regulated by the requirements of functional recovery rather than morphological regeneration. Depending on the characteristics of liver injury, liver regeneration can be classified into two patterns, acute liver regeneration and chronic liver regeneration. Acute liver regeneration is initiated after PHx or a high concentration of chemical injury in a short time, during which all the existing mature cells proliferate to replenish the liver mass. Chemical injury not only causes a regenerative response but also induces an inflammatory response. The degree of liver injury is related to the time and dose of administration. However, chronic liver regeneration is activated by inflammation, viruses, or toxins in continuous time, which yields adverse effects and hinders optimal liver function, leading to cirrhosis and even liver cancer (Forbes and Newsome, 2016; Michalopoulos, 2017; Michalopoulos and Bhushan, 2021). Hepatocyte self-replication mainly contributes to liver regeneration after PHx, while liver progenitor cells (LPCs), which can differentiate into either liver cells or bile duct cells, serve as an alternative pathway for acute regeneration when the tissue injury is too severe to initiate sufficient proliferation of hepatocytes (Li et al., 2020; Zhu et al., 2020). Furthermore, LPCs also appear in hepatic fibrosis and the amount is correlated with the severity of fibrosis in chronic liver diseases (Williams et al., 2014; So et al., 2020).

At present, the protocols for liver injury induction and regeneration mainly include surgical operation and chemical induction (Forbes and Newsome, 2016; Huang et al., 2021). Chemicals are usually toxic to the liver and will cause an inflammatory response, massive necrosis, and high mortality if the dose, frequency, and method of administration are not well controlled. In contrast, PHx serves as the commonly used model in liver regeneration, mainly due to the exact removal of the hepatic mass, the precise timing of stimuli, the minimized hepatocytes damage, and the pure regenerative response without an inflammatory response (Higgins and Anderson, 1931; Michalopoulos and DeFrances, 1997). PHx was primarily invented by Higgins and Anderson in 1931 (Higgins and Anderson, 1931). Since then, this method has been gradually accepted as the commonly used model in rats and a standard procedure has been formed for rodents (Mitchell and Willenbring, 2008). The PHx model completely retains the structure of the main portal vein, inferior vena cava, common bile duct, and hepatic artery, presenting both mechanical and perfusion damage to the liver. It is close to the clinical liver transplantation procedure with valuable experimental results for reference. The characteristics of the liver regeneration model constructed by PHx are as follows: 1) Since a single liver lobe is removed and the process does not lead to a large amount of necrosis of residual liver tissue, the initiation of residual liver regeneration is relatively independent of the inflammatory response, which helps to better explore the direct initiation factors of liver regeneration. 2) The model has good repeatability and stability, and the operation can be completed in approximately 20 min by a skilled surgeon. 3) The regeneration reaction caused by PHx is immediate and can be used as the starting point of the whole regeneration process when the surgery is completed. After PHx, the peaks of DNA synthesis and mitosis in hepatocytes vary depending on different species (24 h in rats and 36–48 h in mice) (Michalopoulos, 2013). Furthermore, PHx has high accuracy in quantifying the degree of liver resection, low complication rate, high surgical success rate, and good repeatability (Forbes and Newsome, 2016; Christ et al., 2021). Based on the above advantages, liver regeneration after PHx is a suitable model to help understand the complexity of signaling pathways for tissue growth, and is widely used in the treatment of clinical liver diseases (*e.g.*, living donor liver transplantation, liver cancer).

Biochemical cues-based liver regeneration theory related to PHx has been extensively investigated, covering the cell proliferation dynamics (Michalopoulos and DeFrances, 1997), the morphologic changes (Michalopoulos, 2007), the extracellular matrix (ECM) reconstruction (Michalopoulos and Bhushan, 2021), and the complete or auxiliary mitogens-mediated signaling pathways (Fausto et al., 2006; Michalopoulos, 2017). Indeed two main signaling pathways, complete mitogens-dependent and auxiliary mitogens-dependent, are found to interact with each other (Taub, 2004). All the events during liver regeneration are finely tuned in time and space (Michalopoulos, 2007). Briefly, the hepatocytes are the first to proliferate with DNA synthesis peaking at 24 h in rats, while DNA synthesis occurs later in the nonparenchymal cells (NPCs). Major complete mitogens include hepatocyte growth factor (HGF) and its receptor c-Met, together with epidermal growth factor (EGF), transforming growth factor-α (TGF-α), as well as heparin-binding EGF-like growth factor (HB-EGF) and their receptor epidermal growth factor receptor (EGFR) (Kimura et al., 2023). Several auxiliary mitogens, such as tumor necrosis factor-α (TNF-α), interleukin-6 (IL-6), and bile acids can orchestrate and optimize the timing and intensity of intracellular signals essential for controlling hepatocyte proliferation and paracrine cell interactions (Michalopoulos, 2013; Michalopoulos, 2017; Tao et al., 2017). These signaling molecules are mainly derived from the paracrine of NPCs, the deposition in ECM and the portal circulation (Michalopoulos and Bhushan, 2021; Shu et al., 2022). After PHx in rodents, liver histology gradually starts to restore at 3–4 days, most of the liver mass is restored within 7–8 days, and the complete restoration is achieved within 3 weeks (Michalopoulos and DeFrances, 1997; Michalopoulos, 2007; Michalopoulos, 2017; Michalopoulos and Bhushan, 2021).

Elucidating those priming factors is one of the hot topics in the field of liver regeneration. There are three broad categories of recognized triggers: biochemical factors, endothelial cell stretch, and sinusoidal shear stress. Studies on the triggering mechanism of liver regeneration after PHx and partial liver transplantation cannot ignore the role of mechanical forces. For example, mechanical stretch on portal vein endothelial cells promotes the release of IL-6 from endothelial cells. After right hepatic portal vein embolization, the diameter of intrahepatic portal vein branches in the residual liver is increased by 150% compared with that in the control group, accompanied by a significant increase in IL-6 secretion (Kawai et al., 2002). Meanwhile, applying the same amount of mechanical stretch to cultured vascular endothelial cells significantly increases the release of IL-6 within 6 h (Kobayashi et al., 2003). On the other hand, after partial liver transplantation or PHx, the volume of the residual liver decreases relatively due to the unchanged total portal blood flow, leading to increased portal perfusion in liver tissue per unit volume and increased portal pressure which results in enhanced shear stress in hepatic sinusoid space. A hepatic sinusoid is a double-barrel structure composed of sinusoid space and Disse space, and hepatocytes are exposed to portal pressure directly through fenestrae on liver sinusoid endothelial cells (LSECs). Therefore, hepatocytes, LSECs, and vascular endothelial cells are all affected by shear stress caused by high portal pressure. Various cell surface receptors found in regulating shear stress on the surface of vascular endothelial cells (Yee and Revel, 1978; Koch and Leffert, 1979) may serve as candidates for liver regeneration. The increased shear stress in the sinusoids due to portal hyperperfusion after PHx or partial liver transplantation may result from the release of regeneration-related factors acting on these receptors. In addition, excessive shear stress after major liver resection leads to liver failure, while the decreased portal shear stress after portal shunt surgery may atrophy the liver until it reaches the new portal pressure and shear stress equilibrium point (Sato et al., 1997; Abshagen et al., 2008). Shear stress also increases the diameter of blood vessels, affects the expression of c-jun, c-myc, c-fos, and other regenerative early genes, and induces the increase of nitrogen oxide synthase (eNOS) expression in LSECs, thereby inducing liver regeneration (Sato et al., 1997; Niiya et al., 1999; Lalor et al., 2006). Recently, hepatocyte heterogeneity has been shown to appear after PHx (Chen et al., 2020), segregating hepatocytes into different functional subgroups based on distinct gene expression patterns including both resting hepatocytes and those with high expression of specific function-related and proliferation-related genes (Walesky et al., 2020; Chembazhi et al., 2021). However, the mechanisms of how resting hepatocytes can transform into a proliferative state and how liver regeneration is initiated still need to be further clarified based on those hemodynamic factors. While attention has been focused on the signaling molecules that promote liver regeneration, the triggers and initiation that induce these changes are not fully understood. Evidently, the mechanism of liver regeneration is extremely complex with highly coordinated proliferative responses of various effector cells, involving several pathways and multiple factors, and the loss of function from a single gene rarely leads to the complete abolition of liver regeneration (Michalopoulos and Bhushan, 2021). Specifically, considering the complex mechanical microenvironments within the liver, mechanical factors may serve as additional candidates playing a direct role in this process. In this review, biomechanical regulation in liver regeneration was discussed specifically in PHx, together with underlying mechanical signaling pathways.

## 2 Mechanical environments in liver regeneration

### 2.1 Hemodynamics in liver regeneration

The liver is a highly vascularized organ that has a unique blood supply. Blood pumped from the heart supplies oxygen to the liver through the hepatic artery, accounting for 1/3 of the total blood volume, while blood from the intestines and spleen converges through the portal vein to metabolize and detoxify substances, accounting for 2/3 of the total volume (Lautt and Greenway, 1987; Eipel et al., 2010). Under physiological conditions, blood pressure in the hepatic artery is close to that of the aorta at approximately 90 mmHg (Balogh et al., 2004; Eipel et al., 2010) but much lower in the portal vein varying from 3 to 10 mmHg (Kumar et al., 2008; Eipel et al., 2010). The blood flow from the portal vein and hepatic artery intersects at the hepatic sinusoids and slowly flows from the edge of the hepatic lobule into the central vein, inferior lobule vein, collecting vein, hepatic vein, and finally into the inferior vena cava. When blood flows into the hepatic sinusoids, where the pressure declines from the periportal to the pericentral region, and the average pressure within sinusoids is estimated to be 1–5 mmHg for the gradient from the portal vein to the inferior vena cava (Kumar et al., 2008; Feng et al., 2014). The hepatocytes take up oxygen from the hepatic arteries and the nutrients brought in by the hepatic portal vein are absorbed, synthesized, and processed in the hepatocytes and the new substances generated are circulated in the body. The blood flow velocity through the hepatic sinusoids is slow, approximately 200–330 μm/s measured by the distance-time image of red blood cells using a two-photon laser scanning microscope, which is positively correlated with low blood pressure within the sinusoids (Cantre et al., 2008; Fan et al., 2019). Low blood pressure and low flow velocity within the sinusoids facilitate the exchange of oxygen, nutrients, and waste products between the circulating blood and the liver (GroSse-Segerath and Lammert, 2021).

On the other hand, 2/3 PHx causes significant perfusion and hemodynamics alterations within the liver remnants, presented on both the macrovascular (portal vein) and the microvascular (sinusoids) scales. After 2/3 PHx, the entire flow needs to traverse through a capillary bed whose cross-section is mathematically down to one-third of the original, a threefold increase in portal vein flow per liver volume (Michalopoulos, 2007; GroSse-Segerath and Lammert, 2021). Compared with those physiological cases, the number of vessels is decreased in the remaining liver and the vessels dilate to allow the same amount of blood to pass through the reduced liver tissues, leading to an increase in portal vein flow rate per liver mass, from 120 to 300 mL/min·g liver tissue (Cantre et al., 2008; Rabbany and Rafii, 2018). In this case, the average blood flow velocity within the hepatic sinusoids increases to 450–500 μm/s in rats and 600 μm/s in mice (Cantre et al., 2008; Marlini et al., 2016; Ishikawa et al., 2021). Intravital fluorescence microscopy reveals that the diameter of sinusoids increases from 6.4 to 7.1 μm in rats after PHx because of the increased pressure within the sinusoids, while liver sections in mice before and 1 h after PHx show a more significant increase in sinusoidal diameter (Lorenz et al., 2018).

A body of evidence indicates that the early hemodynamic changes after PHx are critical, and these alterations induce an overall series of events throughout the entire organ that resembles a wound-healing response (Sato et al., 1997; Michalopoulos, 2007; Yagi et al., 2020). To verify the effect of hemodynamics on liver regeneration after PHx, several methods have been subsequently developed to assess its effect on liver regeneration by altering hemodynamics. The portohepatic shunt (PHS) procedure is targeted at diverting the blood flow surrounding the liver to bypass and split the blood flow directly into the inferior vena cava (Marubashi et al., 2004). Portal pressure and liver weight index are relatively stable in the PHS group compared with the PHx model, illustrating the necessity of the portal hyperdynamic state for liver regeneration. Another method, PHx with partial portal ligation (PHPL) is performed by suturing the vessel to reduce the portal diameter and the blood flow (Balogh et al., 2004). Liver tissue blood flow and liver/body weight recovery ratios are significantly lower in the PHPL group than in the PHx group. There are also some methods to alter hemodynamics without removing the lobes. Portal vein embolization (PVE) is performed to embolize the right portal vein with fibrin glue (Eipel et al., 2010). The diameter of the left anterior portal branch is significantly larger and the volume of the non-embolized hepatic lobe is significantly larger after embolization. The selective portal vein branch ligation (PVL) is the same as those described for PHx, except that those liver lobes are ligated instead of resected (Lautt and Greenway, 1987). Portal venous pressure after PVL increases to the same extent following PHx, which is positively correlated with the shear stress in the liver. These models suggest that decreasing portal venous flow on the basis of PHx attenuates liver regeneration while increasing blood flow by embolization, and ligation has the same effect as liver regeneration (Gock et al., 2011).

Early hemodynamics studies considered increased shear stress to be responsible for the increased portal venous pressure after PHx (Schoen et al., 2001). Unfortunately, the effect of blood flow after PHx is quite complex, and the hemodynamic and biochemical factors in blood cannot be decoupled *in vivo*. The increase of portal blood flow per liver mass also causes an increase in the availability per hepatocyte of biochemical factors derived from the intestine and pancreas. These biochemical factors include EGF and insulin, as well as nutrients derived from the food supply. In this regard, *in vivo* studies done by altering blood flow make it hard to determine whether liver regeneration is induced by hemodynamic effects or the effects of biochemical factors in the blood (Michalopoulos, 2007; Michalopoulos and Bhushan, 2021; Pibiri and Simbula, 2022). To explore whether hemodynamic effects or biochemical factors in the blood are critical for hepatocyte proliferation and liver regeneration, a cell-based mathematical model is developed, which shows that both biochemical factors and hemodynamic effects are important during liver regeneration (Hohmann et al., 2014). Furthermore, complicated hemodynamic changes at the sinusoidal scale can be further translated into mechanical microenvironments acting on the cells within the hepatic sinusoids. Thus, it is important to quantify the mechanical microenvironment within hepatic sinusoids and explore the underlying roles of various mechanical factors in the liver regeneration process by decoupling the complex mechanical environment *in vitro*.

### 2.2 Mechanical environments within hepatic sinusoids during liver regeneration

**FIGURE 1:**
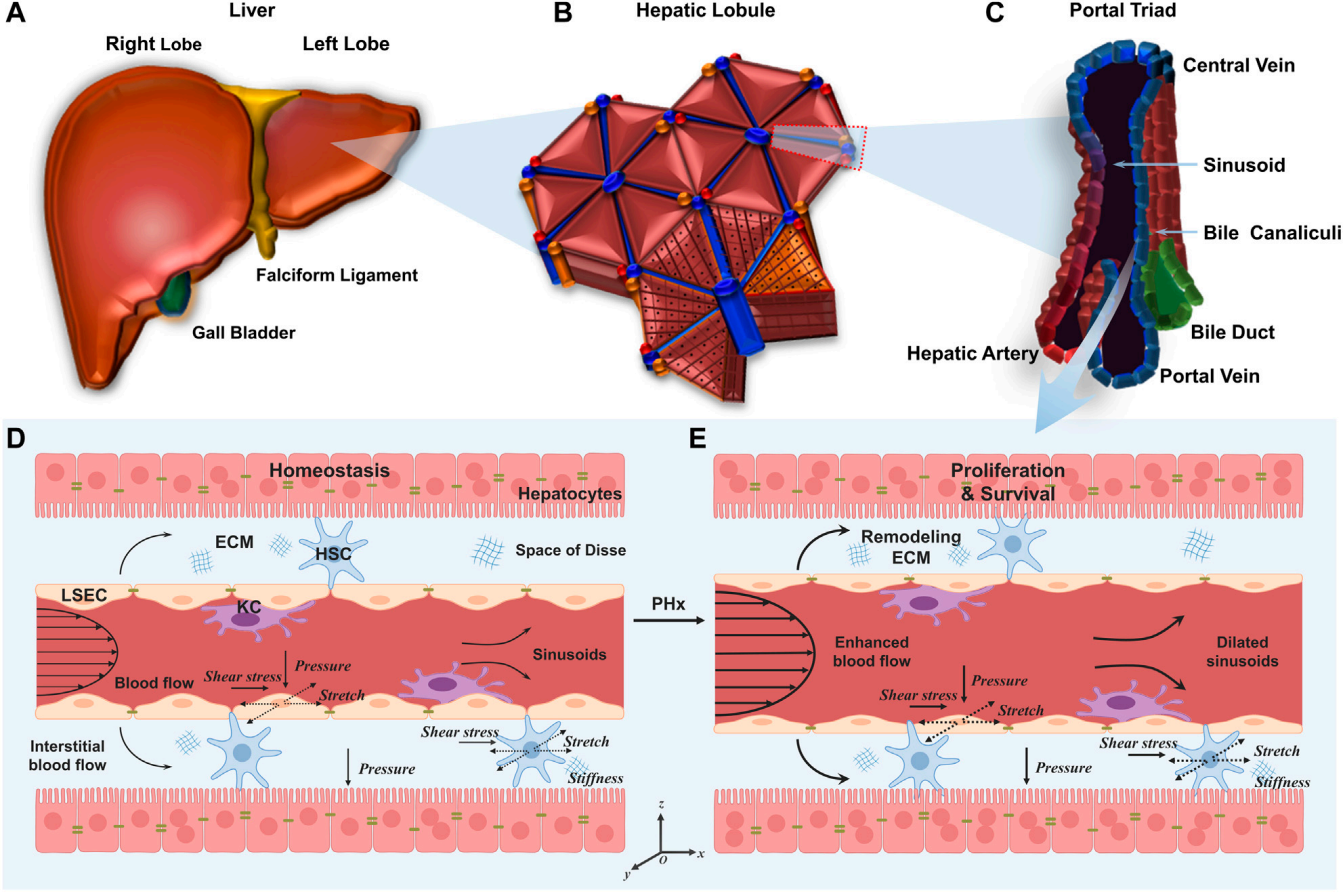
Multiscale microstructures of the liver and its mechanical microenvironment in the normal liver and the liver after PHx. **(A–C)** Plotted are the human liver **(A)**, hepatic lobule **(B)**, and portal triad **(C)**. **(D, E)** Schematics of hepatic sinusoids in the normal liver **(D)** and the liver after PHx **(E)**. In the former, hepatocytes are mitotically quiescent. Physiological blood flow applies shear stress and mechanical stretch on LSECs, and physiological interstitial flow applies shear stress and mechanical stretch on HSCs. In the latter, enhanced blood flow induces high shear stress and stretch on LSECs and HSCs. Those remaining hepatocytes enter the cell cycle to promote cell proliferation.

The liver is composed of different lobes (Figure 1A) that are further divided into numerous hexagonal lobules (Figure 1B), as the basic architectural unit of the liver demarcated by the “portal triad” consisting of the portal vein, bile duct, and hepatic artery. In each lobule, a central vein runs through the lobule center and liver plates formed by hepatocytes are radially aligned (Figure 1C). Hepatocytes constitute 80% of the liver to implement most of the hepatic functions, and the remaining 20% consists of NPCs mainly including LSECs, hepatic stellate cells (HSCs) and Kupffer cells (KCs) (Taub, 2004). The wall of hepatic sinusoids is lined with LSECs. HSCs are in the space of Disse and KCs are in the hepatic sinusoids (Lautt and Greenway, 1987). The existence of permeable fenestrae in sinusoidal endothelium probably enables blood flow to get through the space of Disse underneath the endothelium (Hu et al., 2017). With this complex and dynamic microenvironment, hepatic cells are continuously exposed to mechanical stimuli (Nishii et al., 2018).

Under physiological conditions, the portal vein continues to carry the entire outflow from the intestine, spleen, and pancreas to each hepatic lobule (Figure 1D). As the main cells lining the sinusoids, LSECs are subjected to blood flow that generates two major forces (Rabbany and Rafii, 2018). First, radial blood pressure is applied to the wall of the hepatic sinusoids which, in turn, leads to cyclic stretch and elongation of LSEC layers both perpendicular and parallel to the blood flow, respectively (Anwar et al., 2012). Second, the friction force arising from viscous blood flow over LSEC layers causes axial shear stress (Marubashi et al., 2004). Adjacent HSCs are also likely subjected to cyclic stretch generated by the change in sinusoidal pressure as well as the interstitial blood flow-induced shear stress in the space of Disse (Yi et al., 2015; Wang et al., 2021; Chen et al., 2022). In addition, HSCs persistently produce ECM and the varied ECM stiffness, in turn, manipulates the behaviors of hepatic cells (Long et al., 2022). Finally, hepatocytes are subjected to hydrostatic pressure and interstitial flow within the liver parenchyma (You et al., 2019).

Specifically, after PHx, there are immediate and drastic mechanical changes in the liver (Figure 1E). This hemodynamic change affects the microstructures of hepatic sinusoids, including the increased sinusoidal diameter, the increased blood flow velocity, the enlarged fenestration, the disappearance of sieve-plate appearance, and the widening of inter-cellular spaces, which takes almost 10 days until the entire structure returns to the normal (Morsiani et al., 1998). This vasodilation generates high shear stress and circumferential or axial stretch on LSECs, subsequently inducing the expression and secretion of proteases that degrade and remodel the ECM, thus altering the ECM stiffness and the mechanics of cell-matrix interactions (Song et al., 2017). The threefold increase in portal vein flow also enhances the shear stress and cyclic stretch applied on HSCs in the space of Disse (Rohn et al., 2020). All of these cues suggest that mechanics may serve as one of the earliest events which provide the initiating signals for liver regeneration. Unfortunately, the mechanisms involved in the increased portal vein flow and other mechanical cues as early signals for liver regeneration are not fully understood (Michalopoulos, 2011). Considering the rapid mechanical alterations during liver regeneration, the pronounced changes within hepatic sinusoids, the direct act on LSECs or HSCs, and the fast and direct transcriptional regulation (Tajik et al., 2016) of mechanotransductive signaling, it seems to be particularly important to clarify the roles in differential mechanical cues in liver regeneration after PHx.

## 3 Living donor liver transplantation and mechanical regulation

Liver transplantation is the only effective method and plays an important role in the treatment of end-stage liver disease. However, the shortage of donor livers is always the main problem plaguing its clinical application. A variety of strategies have been developed, including living donor liver transplantation, xenotransplantation, hepatocyte transplantation, increasing the sources of cadaver donor livers, using marginal donor livers, and multiple recipients of one liver. Living donor liver transplantation (LDLT) is highly regarded and has gradually become an effective solution to deal with this challenge. Since the first orthotopic liver transplantation was performed by Starzl in 1969 (Starzl et al., 1969), liver transplantation has developed rapidly. In 1984, reduced-size liver transplantation (RLT) was first proposed to expand the sources of donor livers for pediatric liver transplantation (Bismuth and Houssin, 1984). Since then, the idea of dividing organs, i.e., one liver between two recipients, was proposed to adapt to the increasingly acute disparity between supply and demand of organ transplantation, which laid a theoretical foundation for the development of split liver transplantation (SLT) (Pichlmayr et al., 1988). In 1989, Raia et al. reported the first living donor liver transplant (Raia et al., 1989), and then Strong et al. (Strong et al., 1990) first successfully transplanted the liver of a mother to her son. In 1996, the first adult-to-adult living donor liver transplantation (ALDLT) was performed successfully (Lo et al., 1997). Due to the wide range of applications, the number of LDLTs has increased rapidly in recent years. LDLT involves removing a piece of liver from a healthy donor and giving it to the recipient. Although LDLT has certain deficiencies at present (such as the risk of donor death, the related complications of the donor liver, and the adverse psychological effects on donor and recipient), the advantages of LDLT have been widely recognized by the transplant community. Liver regeneration after LDLT is a precise process involving the proliferative responses of multiple effector cells and the regulation of various cytokines and growth factors. The effects of evident hepatic hemodynamic changes on liver regeneration in the donor and recipient have gradually attracted attention, as discussed below.

### 3.1 Hemodynamic changes of the donor’s liver after PHx and the effect on liver regeneration

Currently, the surgical methods of living donor liver transplantation mainly include left liver transplantation, right liver transplantation, and double liver lobe liver transplantation. In LDLT, the volume of the right donor liver is significantly larger than that of the left donor liver, and the postoperative hemodynamics is altered greatly. Right hemihepatectomy changes the blood circulation of the whole liver, and the pressure and flow velocity inside the portal vein increases correspondingly in the early postoperative period. High perfusion of the portal vein and relatively narrow diameter of the hepatic vein determine the liver function in the early postoperative period. Therefore, various indicators of liver function increase rapidly and reach a peak 1 day after the operation. With the self-regulation of the human body and the gradual stabilization of liver hemodynamics, various liver function indicators gradually return to normal levels within 1 week. Rapid liver regeneration within 2 weeks after surgery is presumably due to the increased liver blood flow and enhanced bile acid absorption (Everson et al., 2013), and appropriately increased portal vein pressure could significantly stimulate the release of factors related to liver blood sinus regeneration, serving as the inducement factor of liver regeneration. Shear stress caused by increased portal blood flow on the vascular wall is conducive to the release of vascular endothelial growth factor, IL-6, and carbon monoxide, thus initiating the regeneration of parenchymal liver cells. Hemodynamic changes in the early postoperative period of the donor are favorable factors for liver regeneration, and the rapid regeneration of the liver further promotes the hemodynamic stability of the entire liver. The early increase in portal vein flow velocity may be attributed to the fact that the liver vascular bed decreases sharply and the blood flow into the liver increases while the total visceral blood flow remains unchanged. The portal vein flow accounts for 75% of the blood flow into the liver, leading to the gradual widening of portal vein diameter to adapt to the changes in liver hemodynamics. With the widening of the portal vein diameter, portal vein flow velocity gradually decreases. After the hepatic artery resistance index decreases sharply in the hepatic vascular bed, the blood flow resistance into the liver increases, and the resistance index increases significantly in the early postoperative period. With the gradual widening of the portal vein and the rapid regeneration of the liver, the blood flow resistance of the liver gradually decreases, and the liver artery resistance index presents a gradual downward trend in the later period. In the process of adapting to the changes in liver hemodynamics, the diameter of the hepatic vein increases accordingly while the velocity of the hepatic vein decreased.

### 3.2 Hemodynamic changes of the recipient’s liver after LDLT and the effect on liver regeneration

After LDLT, the effective vascular bed in the liver is reduced and the blood volume of the entire portal system should be taken over by the remaining partial liver. Therefore, the opening of the portal vein of the transplanted liver faces the problem of excessive perfusion of the portal blood from the graft regardless of the preoperative portal hypertension symptoms. The posterior pulse velocity of patients with cirrhosis is sharply increased after living donor liver transplantation, and the portal blood flow was significantly enhanced after liver transplantation, accompanied by increased peripheral vascular resistance. Compared with non-cirrhotic patients (such as fulminant liver failure and liver tumors), patients with chronic cirrhosis experience a greater increase in backdoor blood flow after transplantation (Piscaglia et al., 1999). Compared with the donor, blood flow in the open back vein of the graft increased significantly (García-Valdecasas et al., 2003). The increased value of portal blood flow and arterial resistance index in the recipient are significantly higher than those in the donor: the portal-arterial blood flow is still placed in a state of dynamic balance. In the early postoperative period, portal blood flow increases significantly, hepatic artery blood flow decreases, and the portal-arterial blood flow balance is broken (Sugimoto et al., 2007). Elevated portal pressure-induced liver regeneration is first identified after hepatectomy or portal embolization. The increased portal pressure after hepatectomy or portal vein embolization leads to increased portal shear stress in the hepatic sinusoids, thus inducing a liver regeneration response. This mechanism has been proven to be an important initiating factor in inducing liver regeneration response in animal experiments (Niiya et al., 1999; Kawai et al., 2002). The rate of early postoperative regeneration in the portal hypertension group was significantly higher than that in the portal hypertension group. Considering the adverse effects of portal hypertension on graft liver function recovery and postoperative survival rate, further studies are needed to define an ideal portal pressure range that is conducive to accelerating liver regeneration without damaging liver function recovery.

## 4 *In vitro* mechanical loading mimicking liver regeneration

The regeneration mechanism is immediately initiated after liver resection. Hepatocyte proliferation compensates for the lost or injured liver tissue and maintains the physiological function of the liver. This process is regulated by liver regeneration factors secreted by liver NPCs, such as LSECs and HSCs. Prior to the changes in the orderly expressions of liver regeneration factors, the portal vein hemodynamics changed significantly due to the increased blood flow sustained by the remaining liver tissues (Sallberg and Pasetto, 2020). However, these changes in hepatic sinusoids caused by increased portal blood flow are complicated as the multiple mechanical factors are coupled together. For example, external forces applied on LSECs can be further translated into the shear stress acting on the surface and the circumferential stretch on the cells within the sinusoids (Morsiani et al., 1998; Shu et al., 2021; Li et al., 2022; Long et al., 2022). To further decouple the contributions of those individual mechanical factors in liver regeneration after PHx, *in vitro* mechanical loading studies have been applied to mimic *in vivo* hepatic sinusoids after PHx and elucidate mechanically-related possible molecular mechanisms, especially in the decoupled forces in the liver microenvironment such as shear stress/stretch.

### 4.1 Shear stress

**FIGURE 2:**
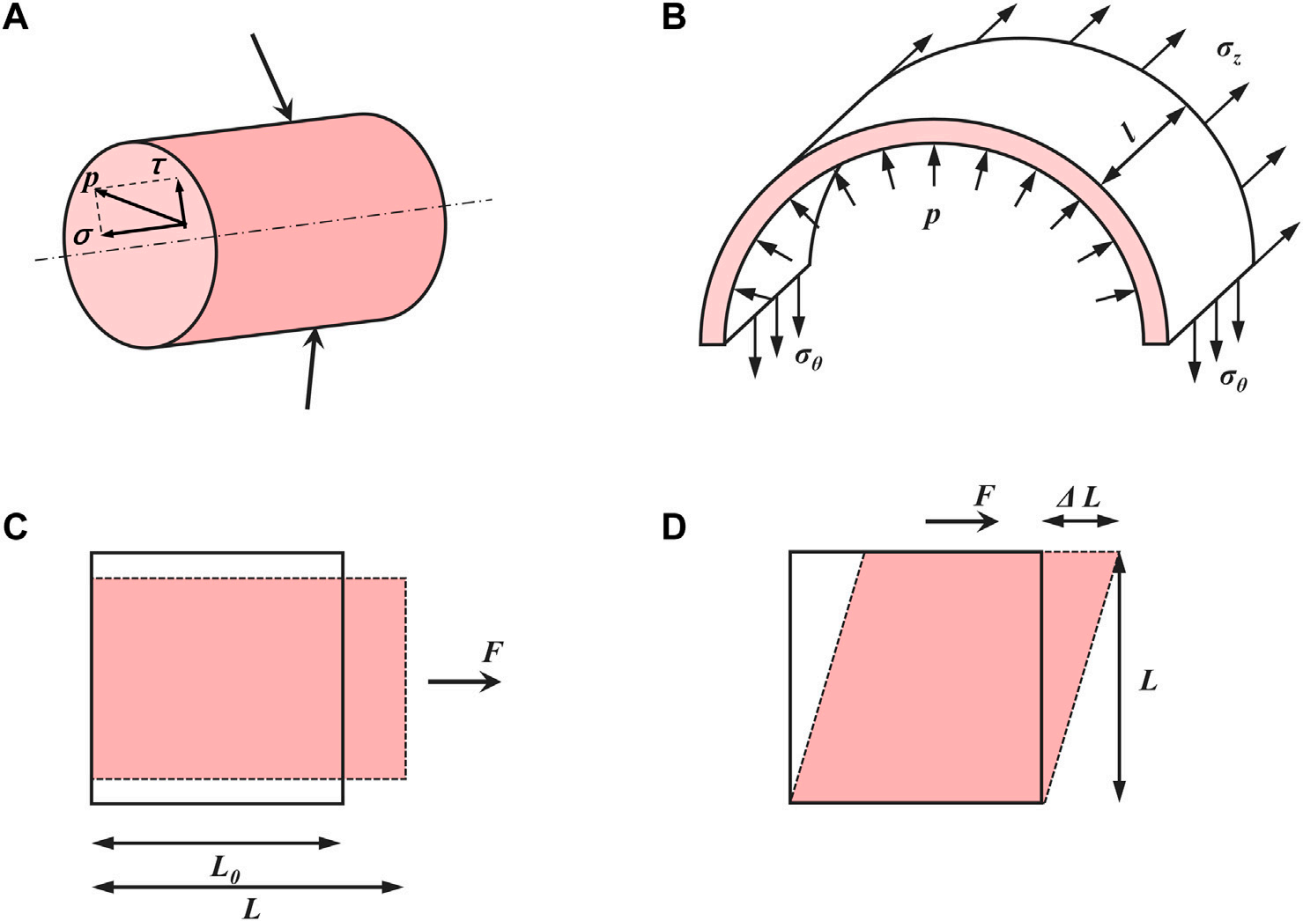
Basic concepts in mechanics of materials. **(A)** Stress (*p*) can be divided into normal stress (*σ*) and shear stress (*τ*). **(B)** The relationship between inner pressure and induced stretch in a thin-walled cylinder. **(C)** Normal strain (*ε*) and **(D)** shear strain (*γ*).

Shear stress (in Pa or dyne/cm^2^), denoted as *τ*, is the shear component coplanar with a material cross-section (Janmey and Miller, 2011) (Figure 2A). Particularly in biology, shear stress is defined as the frictional force generated by the viscous biofluid flow within the lumen of a blood vessel. Within hepatic sinusoids, shear stress is directly caused by blood flow and exerts shear forces on LSECs and adjacent HSCs (Simonetto et al., 2015). LSECs can in turn secrete vasodilators such as NO that affect HSCs within the space of Disse to regulate blood flow (DeLeve et al., 2008; Fernandez, 2015). To date, the exact shear stress within hepatic sinusoids or the space of Disse *in vivo* has not been measured directly and accurately in human or animal models due to the tiny scale and varied sizes of the hepatic sinusoids as well as the vascular permeability induced by LSEC fenestrae (Poisson et al., 2017; Rohn et al., 2020).

Various *in vitro* loading techniques have been developed to study the cellular responses and mechanisms under shear stress. A parallel-plate flow chamber is an ideal device to apply dynamic shear stress *in vitro*, in which the flow is generated through a media reservoir and a peristaltic pump to mimic the *in vivo* environment (Wang et al., 2019). Shear stress in the parallel-plate flow chamber is calculated using the following formula (Ahsan and Nerem, 2010):

$$\tau =\frac{6\mu Q}{bh^{2}}$$ (1)

where *μ* is the blood viscosity (dyne·s/cm^2^), *Q* is the volume flow rate (cm^3^/s), which refers to the volume of fluid passing through a certain cross-section of the blood vessel per unit time, *h* is the chamber channel height (cm) and *b* is the chamber width (cm).

LSECs are the main cell type affected by the changes of shear stress after PHx within sinusoids (De Rudder et al., 2021). For example, a significant increase in the accumulated vasodilators nitric oxide (NO) is presented when primary rat LSECs are exposed to a laminar flow at 14.1 dyne/cm^2^ for 30 min (Shah et al., 1997). This is consistent with the rapidly enhanced secretion response using a bioreactor for real-time NO production at 3 dyne/cm^2^ for 24 h on primary rat LSECs (Illa et al., 2014). This shear stress-induced NO release reinforces the sensitivity of hepatocytes against HGF and therefore triggers the liver regeneration cascade (Wang and Lautt, 1998; Schoen et al., 2001; Golse et al., 2013). In other endothelial cell models, those mechanosensors such as caveolae and ion channels can activate various signaling cascades to regulate NO production by eNOS, suggesting that the response of LSECs may also promote liver regeneration via eNOS-dependent NO secretion (Abshagen et al., 2008; Mei and Thevananther, 2011; Song et al., 2017). Furthermore, inhibiting inducible nitric oxide synthase (iNOS) severely suppresses liver regeneration after PHx in mice (Rai et al., 1998). Laminar shear stress in primary rat LSECs at 14.1 dyne/cm^2^ increases endothelium-specific transcription factor Kruppel-like factor 2 (KLF2) mRNA expressions (Gracia-Sancho et al., 2011; Marrone et al., 2013), which then induces the expression of eNOS, consolidating those KLF2-eNOS-NO signaling mediated by shear stress. However, increased KLF2 expression can also induce an anti-proliferative secretome, which attenuates liver regeneration (Manavski et al., 2017). Moreover, vascular endothelial cell growth factor receptor-2 (VEGFR-2) is responsive to laminar shear stress by translocating from perinuclear distribution to membrane and cytoskeletal localization at 10 dyne/cm^2^ for 15 min with co-localized VE-cadherin (Braet et al., 2004). In the early phases of liver regeneration, VEGFR-2-Id1-mediated inductive angiogenesis in LSECs, through the release of angiocrine factors Wnt2 and HGF, provokes hepatic proliferation, and subsequently, VEGFR-2-Id1-dependent proliferative angiogenesis reconstitutes liver mass (Ding et al., 2010). Transforming growth factor-β1 (TGF-β1), which is known as an inhibitor of hepatocyte proliferation, is observed in a markedly decreased concentration in the culture medium when primary rat LSECs are exposed to a laminar flow at 15 dyne/cm^2^ for 24 h. At the same loading condition, an increase in the intracellular Ca^2+^ level and the phosphorylation status of Erk1/2 are observed after shear stress, indicating that LSECs have the ability to sense shear stress, which in turn induces TGF-β1 production through the G-protein-coupled receptors (GPCRs)-MAPK axis (Hu et al., 2014; Ishikawa et al., 2021). Shear stress not only has a role in the initiation of liver regeneration and the decrease in the late stage of liver regeneration but also induced cellular senescence to blunt liver regeneration (Duan et al., 2022). Genes associated with senescence, such as P16, P53, P21 Pai1, and Gata4 had an upregulated expression when LSECs are subjected to flow conditions with 7.05 dyne/cm^2^ shear stress compared with 14.1 dyne/cm^2^ shear stress, which is mediated by Notch activation. In addition to *in vitro* cell loading, *in vitro* microarrays have also been used to study the process of liver regeneration. A three-dimensional platform called structurally vascularized hepatic ensembles for analyzing regeneration was established to model multiple aspects of human liver regeneration. Exposure of endothelium-lined channels to fluid flow increases the secretion of liver regeneration-associated factors such as HGF as well as cell-cycle entry of primary human hepatocytes embedded within the device (Chhabra et al., 2022). To some extent, the role of fluid shear depends on its magnitude as the excessive increases in shear stress could be detrimental, contributing to stunted liver growth via the release of hepatocyte growth-inhibiting signals and leading to suboptimal liver regeneration (Lorenz et al., 2018).

Not only LSECs but also HSCs are affected by varied shear stresses since there is a permeable flow in the space of Disse across the porous endothelium from the mainstream. For example, primary rat HSCs that are exposed to laminar pulsatile flow ranging from 2.9 dyne/cm^2^, 15 dyne/cm^2^ to 29 dyne/cm^2^ at 2.5 Hz for 1 h present increased HGF mRNA expression and the enhanced HGF secretion into the medium, consistent with a reduction of those matrix-bound pro-HGF proteins after applying shear stress (Rohn et al., 2020). Meanwhile, the impaired mechanosensing via α5β1 integrin in HSCs that contributes to the reduction of HGF release indicates that α5/β1 integrin is an important mechanosensor in HSCs involved in shear-induced liver regeneration.

**FIGURE 3:**
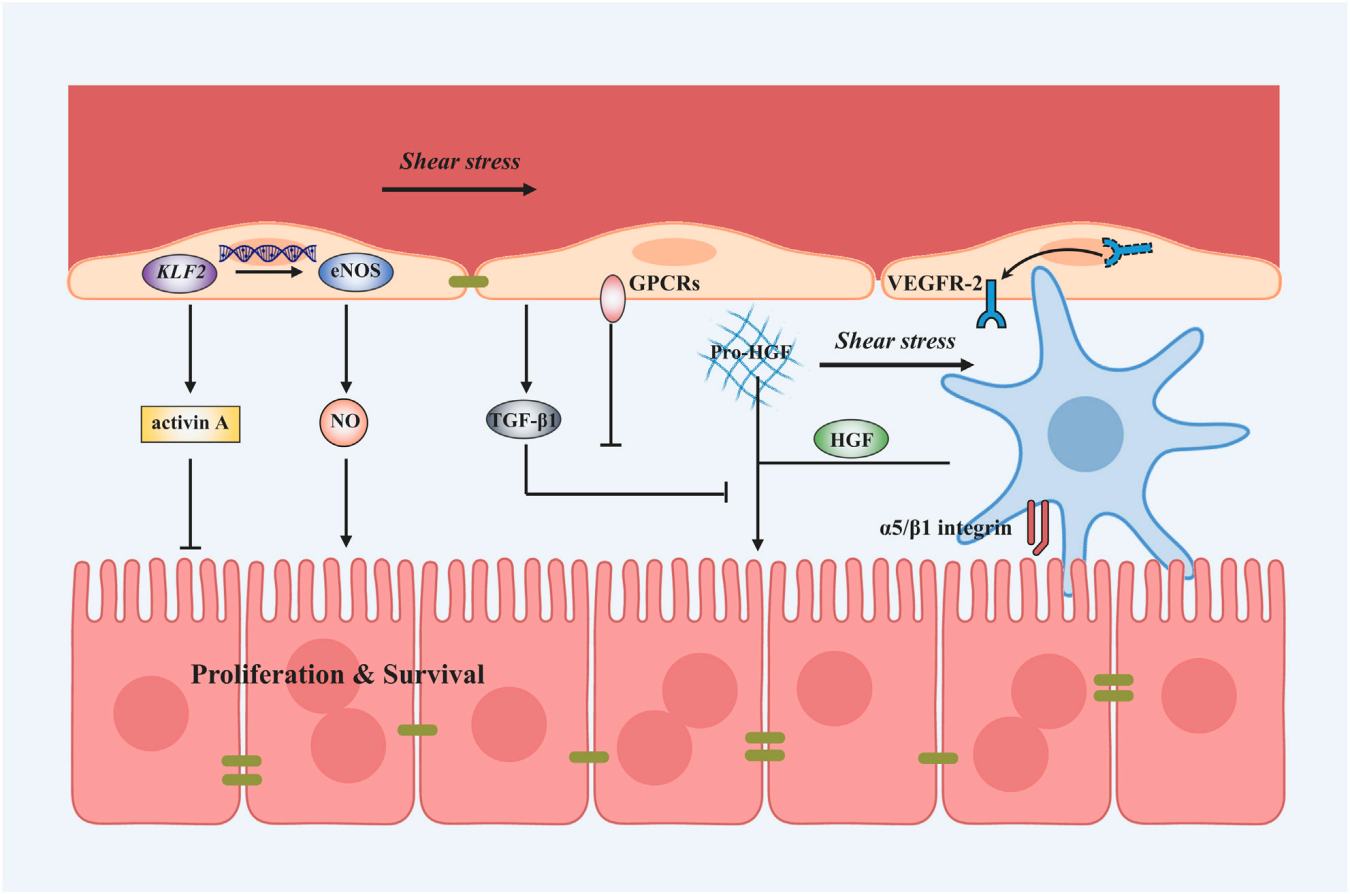
Shear stress-induced mechanotransduction signals in liver regeneration after PHx. Applying shear stress upregulates KLF2 and thus the expression of eNOS and NO. Shear stress can also downregulate TGF-β1 secretion to promote the process of liver regeneration mediated by GPCRs. Besides, VEGFR-2 is translocated to the plasma membrane and might induce angiocrine signals. Shear stress application also activates HSCs to secrete HGF by α5/β1 integrin. A vast amount of dissolved matrix-bound pro-HGF proteins tend to enter the blood flow after shear stress.

Collectively, shear stress can promote liver regeneration by elevating the release of NO and decreasing the secretion of TGF-β1 in LSECs. It can also stimulate liver regeneration by increasing the secretion of HGF in HSCs (Figure 3). This process is referred to as mechanocrine signaling, where changes in mechanical forces are transduced into the secretion of angiocrine signals that affect neighboring cells (Hilscher et al., 2019; Soydemir et al., 2020). NO secreted by LSECs can not only regulate liver regeneration but also relax the vessel through a negative feedback loop. Loading parameters and cellular responses after shear stress are shown in Table 1.

**TABLE 1:** Summary of typical mechanical stimuli and cellular responses during liver regeneration.

Mechanical cues	Cell types	Modes	Parameters	Testing indexes	References
Shear stress	Primary rat LSECs	Laminar flow	14.1 dyne/cm^2^, 30 min	NO	Shah et al. (1997)
	Primary rat LSECs	Laminar flow	3 dyne/cm^2^, 24 h	NO	Illa et al. (2014)
	Primary rat LSECs	Laminar flow	14.1 dyne/cm^2^, 24 h	eNOS, KLF2	Marrone et al. (2013)
	Primary rat LSECs	Laminar flow	14.1 dyne/cm^2^, 12 h	KLF2	Gracia-Sancho et al. (2011)
	Primary rat LSECs	Laminar flow	10 dyne/cm^2^, 15 min	VEGFR-2	Braet et al. (2004)
	Primary murine LSECs	Laminar flow	15 dyne/cm^2^, 3 d	TGF-β1, GPCRs	Braet et al. (2004)
	Primary murine LSECs	Laminar flow	7.05 dyne/cm^2^, 14.1 dyne/cm^2^, 24 h	P16, P53, P21 Pai1, and Gata4	Duan et al. (2022)
	Primary rat HSCs	Pulsatile flow	2.9 dyne/cm^2^, 15 dyne/cm^2^, 29 dyne/cm^2^, 2.5 Hz, 1 h	HGF α5/β1 integrin	Rohn et al. (2020)
Stretch	Primary human LSECs	Uniaxial stretch	50%, 1 Hz 6 h 12 h 24 h 48 h	Length, IL-6, HGF, TNF-α	Kawai et al. (2002)
	Primary human LSECs	Uniaxial stretch	20% constant, 30 min and 20%, 0.5 Hz, 1 h	Length, activated β1 integrin, p-VEGFR-3, HGF, IL-6, TNF-α, MMP-9	Lorenz et al. (2018)
	Primary murine LSECs	Cyclic biaxial stretch	20%, 1 Hz, 4 h	IL-6	Hilscher et al. (2019)
	Primary rat HSCs	Uniaxial stretch	30%, 1 h	HGF	Rohn et al. (2020)
	Primary human and murine HSCs	Cyclic biaxial stretch	10%, 0.5 Hz, 24 h	Fibronectin fibril assembly	Simonetto et al. (2015)
	LI90 cell lines	Cyclic biaxial stretch	10%, 0.5 Hz, 24 h	MMP-1 MMP-2, TIMP-l and TIMP-2	Goto et al. (2004)

### 4.2 Mechanical stretch and pressure

Mechanical stretch is another major hemodynamic force originating from blood flow and is applied on the vessel lumen. In hepatic sinusoids, the stretch is mainly caused by the pressure of blood flow and is applied on LSECs as well as adjacent HSCs (Simonetto et al., 2015). After PHx, the increase in blood flow causes circumferential as well as axial vessel wall expansion, resulting in stretching LSECs and other cell types along the vessel wall (Figure 2B). These lined cells sense the cyclic strain (*ε*) (Figure 2C) in the direction of stretch, defined as (Charras and Yap, 2018):

$$\varepsilon =\left(L-L_{0}\right)/L_{0}$$ (2)

Here the endothelium has an original length *L*_*0*_ and is stretched to a length *L* by the tension acting perpendicular to its surface. Based on the changes in hepatic sinusoidal diameter after 2/3 PHx, the strain on the LSECs after LSECs is estimated to be 10%–20% (Lorenz et al., 2018). The shear strain (*γ*) (Figure 2D) is defined as the deformation in the direction of the shear force divided by the original length perpendicular to it (Charras and Yap, 2018):

$$\gamma =\Delta L/L_{0}$$ (3)

Commercialized Flexcell tension systems and STREX cell-stretching devices are usually used to apply mechanical stretch *in vitro*. Here, cells are seeded on silicone membranes and subjected to cyclic stretch for a period of time with proper strain amplitude and frequency (Zhang et al., 2021).

To examine if cyclic stretch applied to LSECs is involved in the regenerative process after PHx, primary human LSECs cultured on an elastic silicone membrane are subjected to a continuous uni-axial stretch at a strain of 50% and 1 Hz, mimicking the percent increase in the diameter of the portal branch after PVE (Kawai et al., 2002). IL-6 secretion is enhanced while TNF-α and HGF secretions remained unchanged with mechanical stretch within 6–48 h. While this is the first attempt to address whether the alterations in mechanical stretch contribute to liver regeneration-associated cytokine releases, this issue received limited attention in the past decades until mechanical stretch created by the passage of blood through the liver was found to activate the signaling pathways that promote the production of angiocrine factors and the proliferation of hepatocytes (Lorenz et al., 2018). A uni-axial stretch at 20% strain for 30 min and immediately afterward at 20% strain, 0.5 Hz for 1 h was applied to primary human LSECs, mimicking the mechanically-induced sinusoids widening during liver growth or regeneration after PHx. Stretch induces the increased secretion of HGF mediated by activated β1 integrin and phosphorylated VEGFR-3. Enhanced IL-6 and TNF-α secretion, as well as increased matrix metalloproteinase-9 (MMP-9) activity, were also found. Furthermore, those supernatants collected from stretched LSECs also promote the proliferation and inhibited the apoptosis of hepatocytes, suggesting that the mechanotransduction alone is sufficient to turn on the angiocrine signals and cause *in vitro* proliferation and survival of human primary hepatocytes. Evidently, stretch patterns (static vs. cyclic, or uni-axial vs. biaxial) and loading parameters (such as magnitude, frequency, and duration) are critical in the mechanotransductive process (Rabbany and Rafii, 2018). A biaxial, cyclic stretch at 20% strain and 1 Hz was applied to mouse primary LSECs, attempting to recapitulate those pulsatile forces induced by congestion in which mechanical stretch was generated by vascular strain and increased intrahepatic pressure likely resulted in a stretch similar to that after PHx (Hilscher et al., 2019). Even different from the cases of liver regeneration, these stretched-mediated outcomes present the upregulated IL-6 or selectin transcriptions and integrin signaling.

Similar to the findings in shear stress, not only LSECs but also HSCs can sense the cyclic strain based on the expanded sinusoidal diameter. Applying a 30% strain stretch for 1 h to rat primary HSCs that mimics high blood flow enhanced HGF release (Rohn et al., 2020). To simulate the intrahepatic pressure-induced stretch during congestion, applying a cyclic uniform stretch at 10% strain and 0.5 Hz for 24 h to human or murine primary HSCs is able to remarkably increase fibronectin expression and fibril assembly, thus varying the matrix stiffness (Simonetto et al., 2015). Meanwhile, applying a 10% strain at 0.5 Hz for 24 h on LI90 cell lines that mimics mechanical stretch induced by increasing portal blood flow, causes increased MMP-1 and decreased MMP-2 and tissue inhibitor of metalloproteinases-1 (TIMP-1) and TIMP-2 production, suggesting that HSCs are activated by mechanical stretch at the early phase of portal hypertension and that the matrix stiffness has changed (Goto et al., 2004).

**FIGURE 4:**
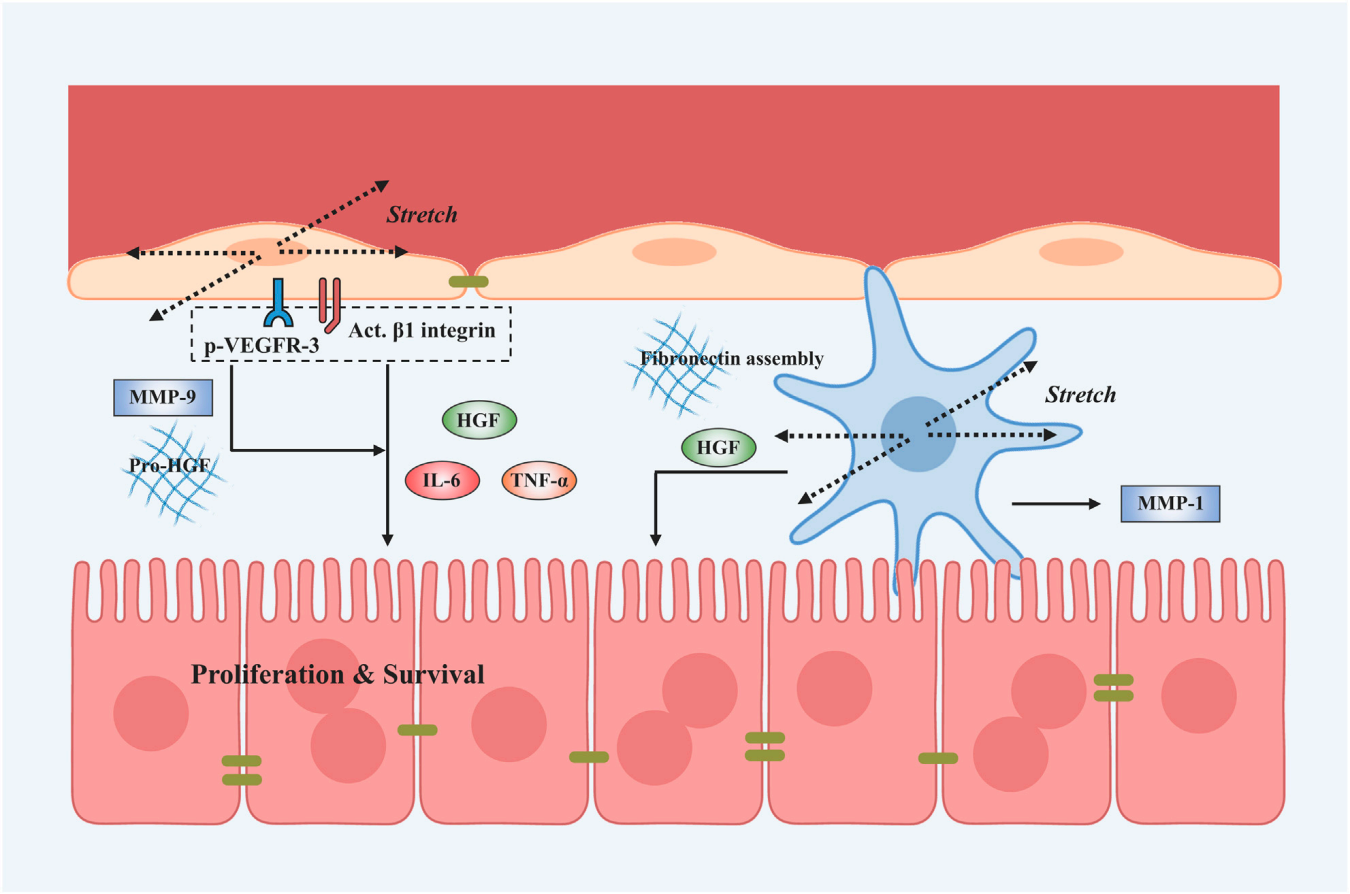
Stretch-induced mechanotransduction signals in liver regeneration after PHx. Applying the stretch activates β1 integrin and its interactions with VEGFR-3 on LSECs. Subsequently, LSECs are able to secrete angiocrine signals such as HGF, IL-6, and TNF-α and activate MMP-9 to stimulate the proliferation and survival of hepatocytes. The stretch application also activates HSCs to secrete HGF and MMP-1 and stimulates fibronectin fibril assembly by HSCs.

Taken together, LSECs and HSCs can respond to mechanical stretch and secrete angiocrine factors that serve as critical regulators of liver regeneration. Existing works usually conflate hemodynamic changes with shear stress, while it is also fundamental that stretching LSECs or HSCs during vasodilation induces angiocrine signals that contribute to liver regeneration (Figure 4). Evidently, angiocrine signals derived from stretching LSECs and HSCs are an important component of intercellular communication and have a key role in organ growth, regeneration, and disease (Marrone et al., 2016). Loading parameters and cellular responses after mechanical stretch are shown in Table 1.

Fluid pressure denotes the hydrostatic pressure exerted outside the blood vessel. Inside the liver, it is applied to LESCs from surrounding hepatic sinusoids and to hepatocytes and HSCs from liver parenchyma. After PHx, the mainstream or interstitial pressure varies transiently with the progress of liver regeneration. From a mechanical viewpoint, this pressure variation is usually coupled with the shear stress and mechanical stretch described above, since the PHx operation can induce simultaneous changes in blood flow, sinusoidal vasodilation, and intrahepatic pressure. Thus, only a few studies have been conducted to isolate the pressure effects on hepatic functions. As an example, placing HepG2 and Huh-7 cell lines under a pressure of 15 mmHg for 24 h significantly increases their proliferation and invasion, with several associated pathways including PI3K-Akt, focal adhesion, integrin, FOXO, and Hippo signaling analyzed from their differentially expressed mRNAs (Shen et al., 2019).

### 4.3 Stiffness

Stiffness is the extent to which a material resists deformation in response to an applied force. It is usually applied to define the rigidity of the tissue in biology. For example, a stiff matrix provides higher resistance than a soft one, as shown by bone vs. liver or a cirrhotic vs. normal liver (Wells, 2008). Atomic force microscopy (AFM) is often applied to determine the stiffness of liver tissues, matrix fiber, or hepatic cells *in vitro*.

Most of the studies that deal with liver stiffness are referred to in the context of liver fibrosis and cirrhosis, whereas the matrix composition and stiffness are also varied during regeneration. After PHx, those elevated expressions of those key molecules such as urokinase plasminogen activator (uPA) and MMP-9 can activate matrix remodeling, and, therefore, release inactive, single-chain HGF bound to the hepatic matrix and change the substrate stiffness (Mueller et al., 2002; Michalopoulos, 2007). Substrate stiffness plays an indispensable role in hepatocyte proliferation, as exemplified by the fact that Huh7 and HepG2 cells cultured on polyacrylamide gel with higher stiffness of 12 kPa resulted in at least a two-fold increase in the cell number compared with cells cultured on a softer substrate of 1 kPa, where substrate stiffness was also measured using AFM (Schrader et al., 2011). A proteomics-based approach for determining the changes in liver ECM composition during liver regeneration reveals that an increase in collagen and a decrease in elastic fibers lead to rearrangement and increased ECM stiffness. These changes regulate hepatocyte proliferation in the regenerating liver (Klaas et al., 2016).

It is well known that liver regeneration is slow after fibrosis or cirrhosis (Xue et al., 2013), but the underlying mechanism is not well understood. After 70% PHx, the level of TNF-α mRNA in the residual liver of healthy rats increases rapidly, reaches a peak at 6 h after PHx, and then decreases slowly. However, the level of TNF-α mRNA in the remaining liver remains quite low at 6–12 h after surgery and then increases slowly until reaching a peak at 24 h after surgery. The peak value is dramatically lower than normal ones and then decreases rapidly. The results of intrahepatic IL-6 mRNA levels of a sclerosed liver also remain quite low at 6–12 h after surgery, and then rise slowly until 72 h after surgery. The peak value is much lower than the normal one and its declination is slow, while the long-term, low level of IL-6 presents an inhibitory effect on liver regeneration (Tiberio et al., 2008). At the same time, STAT3 in the livers of 70% of healthy rats is activated within 30 min after partial resection of the liver and reaches a peak at 3 h after surgery, and the effect lasts for 46 h. Clinical trials have shown that the amount and activity of STAT3 protein in liver tissues of alcoholic cirrhosis and hepatitis cirrhosis are lower than those in normal liver (Horiguchi et al., 2007). The expression of IL-6 and TNF-α is delayed after PHx of the sclerotic liver mentioned earlier, which may be related to the upregulation of Pias3 protein and inhibits STAT3 activity in sclerotic liver (Stärkel et al., 2005). This partly explains the phenomenon that the regeneration of a sclerotic liver starts slowly. Studies have shown that liver fibrosis is not conducive to the regeneration of residual liver after PHx, thus affecting the rapid stability of postoperative liver hemodynamics. Severe fibrosis is a high-risk factor for postoperative liver failure, intractable ascites, and even death of the donor (van den Broek et al., 2008). Early hemodynamic changes, infection, and inflammatory reactions after PHx often lead to acute liver damage. Acute liver injury is often accompanied by an increase in liver stiffness (Dechêne et al., 2010). After PHx, liver stiffness increases transiently in the first week, which might be related to changes in liver hemodynamics, active liver regeneration, and intrahepatic bile stasis (Inoue et al., 2009). Subsequently, liver stiffness gradually decreases and could recover to the preoperative normal level within 5 weeks after surgery.

## 5 Perspectives and conclusion

Liver regeneration after PHx is a sequential process from the beginning of hepatocyte proliferation to the recovery of liver tissue structure. As an abrupt and drastic change within the hepatic sinusoids, the functions and potentials of mechanical cues after PHx should be valued properly. They could serve as the initiating factors and driving forces in liver regeneration and subsequently cause variations in biochemical factors. Moreover, these mechanical loads are directly applied to hepatic cells within sinusoids, inducing fast responses compared to biochemical signals. To date, biomechanical mechanisms in the PHx-induced increase of portal vein pressure and other subsequent mechanical cues, as early signals for initiating liver regeneration, need a more comprehensive understanding. Early basic and clinical *in vivo* studies have shown that biomechanical changes, especially the hemodynamic cues after PHx, promote liver regeneration and propose a coupled pattern of these mechanical cues. In *in vitro* cases, however, hemodynamics in hepatic sinusoids can be decoupled into shear stress and mechanical stretch/pressure along the sinusoidal wall. Complete analysis of mechanical microenvironments based on *in vivo* data is one of the first critical steps to understanding their effects on liver regeneration.

Mechanics can directly manipulate LSECs and HSCs to secrete these liver regeneration-associated factors to promote the proliferation of hepatocytes, *i.e.*, mechanocrine. Different from existing hypotheses of “blood-flow theory” or “hormone theory”, these biomechanical cues emphasize that mechanical signals can promote liver regeneration by modulating the release of biochemical signals. Therefore, biochemical factors and biomechanical cues combine to promote the progress of liver regeneration. More importantly, the patterns and parameters of magnitude, time, and frequency of mechanical loading can specify distinct cellular responses (Wang et al., 2014). It is essential to quantify those mechanical parameters within sinusoids *in vivo* before and after PHx. Meanwhile, *in vitro* coupled loading and cell co-culture can bridge the gap between *in vitro* variable-based mechanical decoupling and the *in vivo* complex mechanical niche. Elucidating these hemodynamic signals in the process of liver regeneration is of great significance for the treatment of liver tumors and liver transplantation related to PHx.

## Figures and Tables

**Table:** 

**AFM**	Atomic force microscopy
**ALDLT**	Adult to adult living donor liver transplantation
**ECM**	Extracellular matrix
**EGF**	Epidermal growth factor
**EGFR**	Epidermal growth factor receptor
**eNOS**	Endothelial nitric oxide synthase
**GPCRs**	G-protein-coupled receptors
**HB-EGF**	Heparin-binding EGF-like growth factor
**HGF**	Hepatocyte growth factor
**HSCs**	Hepatic stellate cells
**IL-6**	Interleukin-6
**iNOS**	Inducible nitric oxide synthase
**KCs**	Kupffer cells
**KLF2**	Kruppel-like factor 2
**LDLT**	Living donor liver transplantation
**LPCs**	Liver progenitor cells
**LSECs**	Liver sinusoidal endothelial cells
**MMP-1**	Metalloproteinase-1
**NO**	Nitric oxide
**NPCs**	Nonparenchymal cells
**PHPL**	PHx with partial portal ligation
**PHS**	Portohepatic shunt
**PHx**	Partial hepatectomy
**PVE**	Portal vein embolization
**PVL**	Portal vein branch ligation
**RLT**	Reduced size live transplantatio
**SLT**	Split liver transplantation
**TGF-α**	Transforming growth factor-α
**TGF-β1**	Transforming growth factor-β1
**TIMP-1**	Tissue inhibitor of metalloproteinases-1
**TNF-α**	Tumor necrosis factor-α
**uPA**	Urokinase plasminogen activator
**VEGFR-2**	Vascular endothelial cell growth factor receptor-2
